# Immunodetection of occult eosinophils in lung tissue biopsies may help predict survival in acute lung injury

**DOI:** 10.1186/1465-9921-12-116

**Published:** 2011-08-26

**Authors:** Lian Willetts, Kimberly Parker, Lewis J Wesselius, Cheryl A Protheroe, Elizabeth Jaben, P Graziano, Redwan Moqbel, Kevin O Leslie, Nancy A Lee, James J Lee

**Affiliations:** 1 Division of Pulmonary Medicine, Department of Internal Medicine, Mayo Clinic Arizona, Scottsdale, AZ 85259 USA; 2 Pulmonary Research Group, Department of Medicine, University of Alberta, Edmonton, Alberta Canada T6G 2S2; 3 Division of Pulmonary Medicine, Department of Biochemistry and Molecular Biology, Mayo Clinic Arizona, Scottsdale, AZ 85259 USA; 4 Department of Laboratory Medicine and Pathology, Mayo Clinic Arizona, Scottsdale, AZ 85259 USA; 5 Department of Immunology, Faculty of Medicine, University of Manitoba, Winnipeg, Manitoba Canada R3E 0W3; 6 Division of Hematology and Oncology, Department of Internal Medicine, Mayo Clinic Arizona, Scottsdale, AZ 85259 USA

**Keywords:** Acute Lung Injury, Acute Respiratory Distress Syndrome, Eosinophils, Eosinophil Peroxidase

## Abstract

**Background:**

Acute lung injury (ALI) is a serious respiratory disorder for which therapy is primarily supportive once infection is excluded. Surgical lung biopsy may rule out other diagnoses, but has not been generally useful for therapy decisions or prognosis in this setting. Importantly, tissue and peripheral blood eosinophilia, the hallmarks of steroid-responsive acute eosinophilic pneumonia, are not commonly linked with ALI. We hypothesized that occult eosinophilic pneumonia may explain better outcomes for some patients with ALI.

**Methods:**

Immunohistochemistry using a novel monoclonal antibody recognizing eosinophil peroxidase (***EPX-mAb***) was used to assess intrapulmonary eosinophil accumulation/degranulation. Lung biopsies from ALI patients (*n = *20) were identified following review of a pathology database; 45% of which (i.e., 9/20) displayed classical diffuse alveolar damage (ALI-DAD). Controls were obtained from uninvolved tissue in patients undergoing lobectomy for lung cancer (*n = *10). Serial biopsy sections were stained with hematoxylin and eosin (***H&E***) and subjected to ***EPX-mAb ***immunohistochemistry.

**Results:**

***EPX-mAb ***immunohistochemistry provided a >40-fold increased sensitivity to detect eosinophils in the lung relative to ***H&E ***stained sections. This increased sensitivity led to the identification of higher numbers of eosinophils in ALI patients compared with controls; differences using ***H&E ***staining alone were not significant. Clinical assessments showed that lung infiltrating eosinophil numbers were higher in ALI patients that survived hospitalization compared with non-survivors. A similar conclusion was reached quantifying eosinophil degranulation in each biopsy.

**Conclusion:**

The enhanced sensitivity of ***EPX-mAb ***immunohistochemistry uniquely identified eosinophil accumulation/degranulation in patients with ALI relative to controls. More importantly, this method was a prognostic indicator of patient survival. These observations suggest that ***EPX-mAb ***immunohistochemistry may represent a diagnostic biomarker identifying a subset of ALI patients with improved clinical outcomes.

## Background

ALI encompasses a spectrum of pulmonary disorders that is often accompanied by life-threatening hypoxemic respiratory failure and diffuse bilateral pulmonary infiltrates. Moreover, the origins of ALI are often complex (e.g., pneumonia, sepsis, or left atrial hypertension) and not easily attributed to a defined cause [1-6]. In its most dramatic clinical form, acute respiratory distress syndrome (ARDS), precipitous impairment of gas exchange is associated with a high mortality rate (38.5%), especially among elderly patients [7]. Therapy for most patients with idiopathic ALI is limited to supportive care and infection prevention [6,8-10]. Select studies have suggested a benefit of corticosteroid therapy in a subset of patients with ALI/ARDS (e.g., [11-14]). However, large prospective studies have not supported a consensus opinion for the routine use of corticosteroids [15-18].

The pathogenesis of ALI remains unclear, largely a consequence of the heterogeneity of patients coming into the ICU and the broad clinical features characteristic of ALI [1,19]. The cellular mechanisms contributing to lung tissue injury in ALI are for the most part unknown,[20] although the potential involvement of neutrophils in the development of ALI remains the focus of many studies (see for example [21,22]). In particular, neutrophil-derived products (e.g., extracellular matrix degrading proteinases and reactive oxygen species), inflammatory fibrotic cytokines, and growth factors [23-26] have been postulated as causative agents underlying the onset and progression of disease [27].

For most patients with ALI, eosinophils have not been reported as a prominent histological feature, despite their potential tissue damaging capability [5,28] and their presence in a number of other well defined pulmonary diseases such as asthma [29] and acute eosinophilic pneumonia [30]. However, among this larger literature there are some studies that have implicated this granulocyte as either a potentially important contributor to disease or, at the least, a diagnostic biomarker of events occurring in ALI patients [21,27,31]. Interestingly, these studies suggested a "predictive" and "discriminative" value of eosinophil activity assessments in designing an effective therapy for patients exhibiting clinical features of ALI. Unfortunately, the lack of an observable eosinophil infiltrate at the histological level remains a significant confounding issue, especially for those patients who may have been treated (even briefly) with systemic corticosteroids. In turn, the typical absence of visible eosinophils in most cases of ALI that come to biopsy has limited the further development of hypotheses and experimental studies investigating a role(s) of eosinophils in ALI.

In this study we demonstrate that standard histological evaluation of lung biopsies significantly underestimates eosinophil accumulation when compared to assessments using a novel eosinophil peroxidase (***EPX***)-specific monoclonal antibody (***EPX-mAb***) visualized by immunohistochemistry. We employed this unique increase in sensitivity to detect eosinophils and evidence of tissue degranulation in a group of ALI patients. A retrospective assessment of ALI patients who had a lung biopsy taken during the course of their acute care was conducted, comparing the results of these assessments with lung tissue from control subjects. These blinded assessments revealed that the added sensitivity of ***EPX-mAb ***immunohistochemistry detected a significant increase in eosinophil accumulation/degranulation in ALI vs. control subjects. More importantly, ***EPX-mAb ***immunohistochemistry identified a subset of ALI patients who survived hospitalization, suggesting its use as a prognostic indicator may represent a previously underappreciated diagnostic strategy in the management of pulmonary patients.

## Methods

### IRB

The patient studies presented in this manuscript were performed in accordance with NIH guidelines and Mayo Foundation institutional policies (Institutional Review Board, 08-001908 Immunohistochemical Study of Eosinophil Degradation in Archived Lung Biopsies), and in compliance with HIPPA guidelines for patient privacy.

### Study Subjects

An overview of the demographic data and pathological/clinical assessments of our ALI study subjects is presented in Tables 1 and 2. These pulmonary patients were initially identified by study personnel from a search of the Mayo Clinic Arizona Pathology Database with the search key words: lung, biopsy, acute lung injury (ALI), diffuse alveolar damage (DAD), organizing pneumonia, and ARDS. Study personnel subsequently reviewed the available pathology reports and clinical medical records to identify the subset of patients who had received a diagnosis of ALI. Thus, to be included in this study a given patient had to have a diagnosis of ALI and have undergone a biopsy during their course of treatment. It is noteworthy, that this process was not discriminatory and all patients with available biopsy material that had received an ALI diagnosis as part of their standard-of-care were included in this study. The ALI patients included in this study met AECC established clinical criteria [4] for an acute lung injury diagnosis and also displayed characteristic pathological changes linked with this disease. Specifically, assessments of all study patients upon admission with acute respiratory distress revealed the presence of diffuse radiographic abnormalities and hypoxia characterized by an A-a gradient (PaO2/FiO2) less than 300 [4,32]. Indeed, the PaO2/FiO2 ratio in a subset of these cases (11 of 20 total cases) was below 150 and thus would meet clinical criteria for ARDS [4,32]. In addition, the twenty ALI patients in this study included thirteen patients (independent of PaO2/FiO2 levels) that required mechanical ventilation during their hospitalization. Review of patient medical records also revealed the absence of typical risk factors associated with ALI, including myocardial infarction, pulmonary embolism, infection/sepsis (e.g., pneumonia), and acute drug reaction. The pathology evaluations of all study-subjects confirmed the clinical indications of ALI. That is, each patient included in our study displayed three specific histopathologies in the available biopsies [33]: ***(i) ***The presence of fibrin in the alveoli; ***(ii) ***The demonstration of an organizing pneumonia (i.e., a prominent airway cellular infiltrate); and ***(iii) ***Evidence of reactive airway epithelial Type II cell hyperplasia. Among these twenty ALI patients, 45% (i.e., 9/20) displayed classical diffuse alveolar damage (ALI-DAD). Seven of the study group did not survive hospitalization, and of these non-surviving patients >71% (5/7) received an ALI-DAD diagnosis. A variety of co-morbid medical disorders were evident in our ALI patients, including several patients with connective tissue disorders. However, examination of the medical records and care-giver notes failed to identify elements of commonality regarding disease onset or progression. Nonetheless, the unresolved character of disease progression in these subjects was such that nineteen of the twenty patients received corticosteroid therapy during their course of treatment.

**Table 1:** Demographic Data on Control and Acute Lung Injury (ALI) Study Subjects

Study Subjects	Age	Smoking History (Pack - Years)	Gender	
			
			Male (M)	Female (F)
Acute Lung Injury	64.0 ± 2.6	21.1 ± 5.3	11	9

Controls	68.4 ± 4.5	35.2 ± 13.6	3	7

**Table 2:** Clinical Characterization of Patients with Acute Lung Injury

Patient	Concurrent Clinical Diagnoses	Gender	Age	**PaO**_**2**_**/FIO**_**2**_	Ventilator	Hospital Death	Pathology Diagnosis
1	Esophageal carcinoma	M	51	80	Yes	Yes	ALI

2	SLE	F	73	290	No	No	ALI

3	Dressler's syndrome	M	72	134	Yes	Yes	ALI

4	Lymphoma	F	63	204	Yes	No	ALI

5	Crohn's Disease	F	63	205	No	No	ALI with possible AEP

6	HIV+	M	64	150	Yes	No	ALI-DAD With PCP

7	Multiple myeloma	F	73	111	Yes	No	ALI-DAD

8	Lung cancer	F	65	100	Yes	No	ALI-DAD

9	Hepatitis C	M	41	89	Yes	Yes	ALI-DAD

10	SLE	F	75	156	Yes	Yes	ALI-DAD

11	MCTD/pulmonary fibrosis	M	65	93	Yes	Yes	ALI-DAD

12	Dematomyositis	F	42	149	No	Yes	ALI-DAD

13	SLE	M	77	200	No	No	ALI

14	Pneumoconiosis	M	69	66	Yes	No	ALI

15	Drug-induced lung toxicity	M	61	223	No	No	ALI with fibrosis

16	Esophageal carcinoma	M	67	96	Yes	No	ALI-DAD

17	RZ/possible aspiration	F	72	205	No	No	ALI

18	CVD	F	41	167	No	No	ALI-DAH

19	Goodpasture syndrome	M	72	133	Yes	Yes	ALI-DAD with HSV infection

20	Wegener syndrome	M	73	205	No	No	ALI with fibrosis

**Table Abbreviations**: AEP- Acute Eosinophilic Pneumonia; ALI Acute- Lung Injury; ALI-DAD- Acute Lung Injury which includes Diffuse Alveolar Damage; ALI-DAH- Acute Lung Injury which includes Diffuse Alveolar Hemorrhage; CVD- undifferentiated Collagen Vascular Disorder; DAD Diffuse Alveolar Damage; DAH- Diffuse Alveolar Hemorrhage; HIV Human Immunodeficiency Virus; MCTD- Mixed Connective Tissue Disorder; PCP Pneumocystis Pneumonia; RA- Rheumatoid Arthritis; SLE Systemic Lupus Erythematosus

Control lung samples consisted of uninvolved areas of lung tissue recovered from patients undergoing resection for a diagnosis of lung cancer (Table 1). None of the control subjects were receiving systemic corticosteroids, although four control subjects were receiving inhaled corticosteroids.

### Recovery and processing of bronchial tissue biopsies

The twenty lung specimens obtained from patients with ALI included sixteen surgical lung biopsies and four transbronchial lung biopsies considered adequate for histologic review (i.e., containing alveolated lung parenchyma). All control biopsies were taken from uninvolved areas of surgically resected lung tissue removed for treatment of lung cancer. Lung tissue biopsies were fixed in 4% buffered formalin, embedded in paraffin, and serial 4 μm thick sections were cut.

### Histopathological and immunohistochemical staining of lung tissue sections

H&E staining was performed using an automated stain processing unit in the Mayo Clinic Arizona clinical histology unit. Immunohistochemistry was performed using a ***EPX-mAb ***as previously described [34]. Evaluations of the slides were performed with either an Olympus BX50 or a Zeiss Axiophot compound microscope.

### Histopathological evaluation of patients and the quantification of **EPX-mAb **immunohistochemistry

Serial sections from each biopsy were coded by clinical histopathology laboratory personnel and in each case the middle (slide 2) of the three serial sections was stained with Hematoxylin - Eosin (H&E). Slide 1 of the series was subjected to immunohistochemical staining with ***EPX-mAb ***and slide 3 served as an isotype immunoglobulin negative control for the immunohistochemistry.

The eosinophil infiltration of lung tissue using H&E staining was performed in an investigator-blinded fashion independently by an experienced pulmonary pathologist with a specialty in lung diseases (KL) and a pathology resident (PG). The slides were evaluated by each individual as a numerical average calculated from 10 randomly selected hpf (high powered fields; 40x objective/10x ocular lens, 0.29 mm^2 ^field of view); that is, a total area of ~3 mm^2 ^per biopsy-investigator. The reported values are the mean ± SEM of all investigator-derived counts.

Quantification of eosinophil tissue infiltration within each biopsy using ***EPX-mAb ***immunohistochemistry was independently performed in an intra/inter-blinded fashion that included investigators from pathology (KL, EJ, and PG - ***EPX-mAb ***based eosinophil counts in lung tissue); KL and EJ - ***EPX-mAb ***assessments of eosinophil degranulation), a hospital/clinic-based pulmonary fellow (KP - ***EPX-mAb ***based eosinophil counts in lung tissue), and a PhD graduate research fellow (LW - ***EPX-mAb ***based eosinophil counts and degranulation in lung tissue). All evaluations were done at a magnification of 400x. The number of positively stained eosinophils was determined in the alveolar lung parenchyma as a numerical average calculated from 10 randomly selected hpf; that is, a total area of ~3 mm^2 ^per biopsy-investigator. The reported values are the mean ± SEM of investigator-derived counts.

The level and extent of eosinophil degranulation observed within each patient biopsy was also determined by scanning 10 randomly selected hpf (40x objective/10x ocular lens, 0.29 mm^2 ^field of view); that is, a total area of ~3 mm^2 ^per biopsy-investigator. Each field examined was graded using a scale that permitted stratifying the available patients based on a relatively low resolution grading scale that was easily reproduced by multiple evaluators of varying levels of experience and expertise: Level 0 = No identifiable eosinophils and/or degranulation [35]; Level 1a = The field shows evidence of eosinophil degranulation (i.e., extracellular release of EPX) that represents ≤10% of the field's total area and has <3 independent areas within the field displaying degranulation; Level 1b = The field shows similar evidence of eosinophil degranulation as Level 1a but instead displays ≥3 independent areas within the field with evidence of degranulation; Level 2a = The field shows evidence of eosinophil degranulation that includes extracellular release of EPX, enucleated eosinophils (i.e., cytoplasmic fragments), and/or the presence of free eosinophil granules. The extent of degranulation represents 10 - 50% of the field's total area; Level 2b = The field shows similar evidence of eosinophil degranulation as Level 2a but instead has a level of degranulation representing >50% of the field's total area. Eosinophil degranulation was quantified for each of 10 randomly selected hpf of a given biopsy (i.e., patient) by initially applying an increasing numerical value to the level of degranulation evident in the field (Level 0 = 0, Level 1a = 1, Level 1b = 2, Level 2a = 3, and Level 2b = 4). The extent of eosinophil degranulation in the biopsy was then determined as the average of the numerical values assigned to each of the 10 hpf examined. This grading of eosinophil degranulation was performed independently by three outcome-blinded evaluators (an experienced pulmonary pathologist (KL), a pathology resident-fellow (EM), and a Ph.D. graduate student (LW)), all of whom were also unaware of the scores reported by the other evaluators. Degranulation scores are reported as the mean numerical value derived from all three evaluators ± SEM.

### Statistical Analysis

Data are expressed as the mean ± SEM. Statistical analysis for comparisons between groups was performed using either a Student's T test or a Wilcoxon Two-Sample Test for non-parametric data for comparisons between data sets that were not uniformly distributed. Differences between mean values were considered significant when p < 0.01. Intraclass correlation coefficients (ICC) were also determined between investigators reading slides as a measure of inter-rater agreement [36].

## Results

### EPX-mAb immunohistochemistry provides an enhanced level of sensitivity for the detection of eosinophils infiltrating lung biopsies

**Figure 1:**
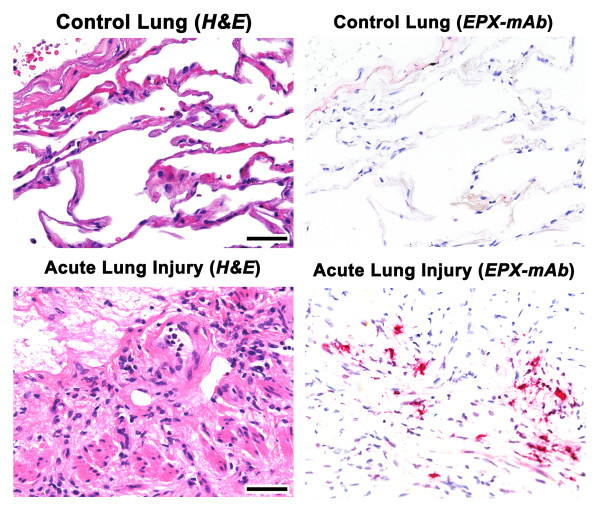
***EPX-mAb *immunohistochemistry represents a sensitive and novel strategy relative to *H&E *staining for the detection of infiltrating eosinophils as well as evidence of eosinophil degranulation in the pulmonary parenchyma**. Side-by-side comparisons of serial lung sections stained with ***H&E*** and sections subjected to  ***EPX-mAb***immunohistochemistry (red staining cells and extracellular matrix areas) are presented from control subjects and an ALI patient. Scale bar = 50 μm.

**Figure 2:**
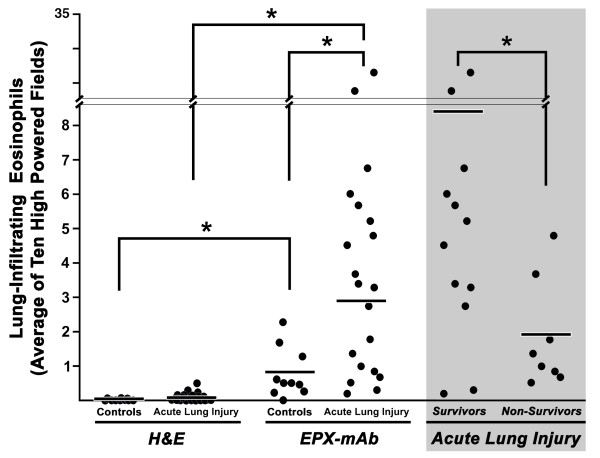
**Assessment of individual patient biopsies revealed that unlike traditional H&E histopathology, *EPX-mAb *immunohistochemistry demonstrated that ALI patients have increased levels of eosinophils relative to control subjects and that within the ALI cohort this increase correlated with patient survival**. Serial sections from either control subjects or acute lung injury patients were stained with ***H&E ***and subjected to ***EPX-mAb ***immunohistochemistry prior to evaluation for infiltrating eosinophil numbers per high powered field. Eosinophil counts per hpf were determined by individual investigators (*n = *2) as the average count resulting from the examination of 10 randomly selected fields; investigators were blinded to both the clinical outcome and the scores of the fellow evaluator. The scatter plots presented represent values for each individual patient derived as the mean of the average eosinophil counts from these evaluators (ICC = 0.785 (95% confidence interval: 0.540 to 0.908). The scatter plots within the shaded area represent acute lung injury patients following ***EPX-mAb ***immunohistochemistry that were then stratified (following decoding of the data) on the basis of their hospital survival. The mean for each cohort is presented as a horizontal bar. *p < 0.01

Serial biopsy sections from either control or ALI subjects were stained for evaluations of eosinophil tissue infiltration. Representative photomicrographs of ***H&E ***stained slides as well as lung sections subjected to ***EPX-mAb ***immunohistochemistry are shown in Figure 1. The quantitative evaluations of eosinophil density using both staining methods are presented as individual patient assessments in the histograms of Figure 2. As expected, the evaluation of the ***H&E ***stained patient biopsies revealed little evidence of eosinophil infiltration in both control and ALI subjects (0.02 infiltrating eosinophils/*hpf *and 0.04 infiltrating eosinophils/*hpf*, respectively). However, evaluation by the same pathology investigators of the serial slides subjected to ***EPX-mAb ***immunohistochemistry demonstrated an enhanced sensitivity to detect eosinophils in these lung tissue sections. Specifically, evaluations of the lungs of control subjects using ***EPX-mAb ***immunohistochemistry revealed a >40-fold increase in the ability to detect tissue infiltrating eosinophils relative to ***H&E ***staining (0.81 eosinophils/*hpf vs*. 0.02 eosinophils/*hpf*, p < 0.01).

### Eosinophil infiltration of the pulmonary parenchyma is higher in ALI patients compared to control subjects and is a diagnostic indicator of patient survival

Evaluation of serial lung sections following ***EPX-mAb ***immunohistochemistry demonstrated that the density of pulmonary eosinophils in the collective group of ALI patients is significantly higher relative to control subjects (3.6-fold, 2.88 eosinophils/*hpf *vs. 0.81 eosinophils/*hpf *(p < 0.01), respectively). More importantly, further evaluations of the ALI patients (Figure 2, shaded histograms) surprisingly showed that ***EPX-mAb ***detection of infiltrating lung eosinophils divided these patients into subjects which survived *vs*. those that did not survive hospitalization (8.4 ±2.9 eosinophils/*hpf vs*. 1.9 ± 0.6 eosinophils/*hpf*, p < 0.01).

### ALI patients surviving hospitalization display significant levels of eosinophil degranulation (i.e., extracellular matrix deposition of EPX) compared with non-surviving patients

**Figure 3:**
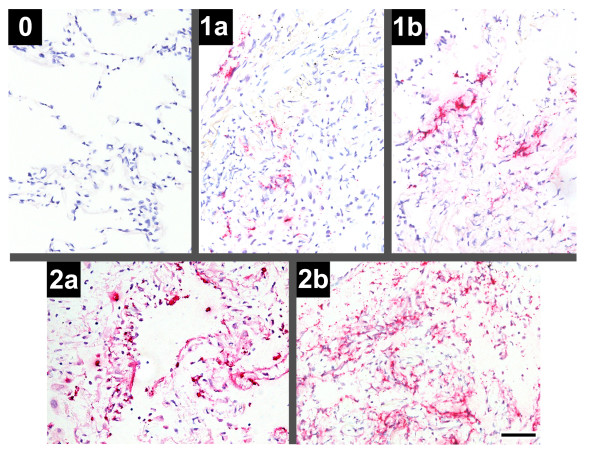
**Acute Lung Injury patients display quantitatively different levels of eosinophil degranulation that may occur even in the absence of intact infiltrating eosinophils**. Representative photomicrographs of the five described levels of eosinophil degranulation within biopsies from ALI patients. **Level 0**: No evidence of eosinophil degranulation. **Level 1a**: Nominal levels of eosinophil degranulation representing <3 areas of granule protein release that is <10% of the field of view. **Level 1b**: Slightly elevated level of eosinophil degranulation representing ≥3 areas of granule protein release that again is <10% of the field of view. **Level 2a**: Significant level of eosinophil degranulation that includes 10-50% of the field of view. **Level 2b**: Significant level of eosinophil degranulation that includes extracellular release of EPX, enucleated eosinophils (i.e., cytoplasmic fragments), and/or the presence of free granules (i.e., EPX-containing secondary granules not associated with fragmented eosinophils). The extent of degranulation represents **>50% **of the field's total area. Scale bar = 50 μm.

**Figure 4:**
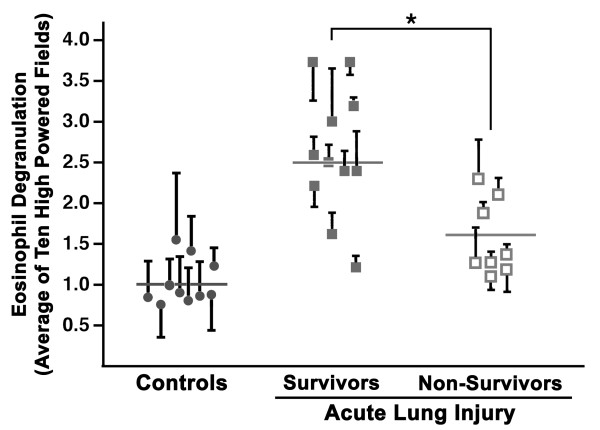
***EPX-mAb *immunohistochemistry provides a quantitatively significant strategy to distinguish acute lung injury patients that survive their hospitalization *vs*. those patients that did not survive**. Sections from acute lung injury patients were subjected to ***EPX-mAb ***immunohistochemistry prior to evaluation for evidence of eosinophil degranulation as described in the Materials amd Methods and the legend of Figure 3. Eosinophil degranulation scores were determined by individual investigators (*n = *3) as the average numerical score resulting from the examination of 10 randomly selected high powered fields (*hpf *- 400x); investigators were blinded to both the clinical outcome and the scores of the fellow evaluator. The scatter plots presented represent values for each individual ALI patient stratified based on hospital survival. Patient eosinophil degranulation values are expressed as the mean of the average eosinophil degranulation score from all three evaluators. The error bars associated with each patient data point is the SEM linked with the mean value derived from each of the three evaluators. The mean for each cohort is presented as a horizontal bar. *p < 0.01

Assessments of lung sections following ***EPX-mAb ***immunohistochemistry revealed that ALI patients displayed significant and varying levels of degranulation that were quantifiable. This degranulation was often observed in these patients in the absence of identifiable intact eosinophils. The photomicrographs of Figure 3 are representative of the stratified levels of increasing degranulation observed in ALI patients from no evidence of degranulation (**Level 0**) in a given high powered field to >50% of the field evidencing eosinophil degranulation (**Level 2b**). Similar to the higher levels of eosinophil infiltration observed in the collective group of ALI patients, the collective group also evidenced a >2-fold increase in the level of eosinophil degranulation compared to control subjects (2.20 ± 0.15/*hpf vs*. 1.02 ± 0.38/*hpf*, respectively). More importantly, quantitative assessments of degranulation (i.e., mean numerical score ±SEM) based on ***EPX-mAb ***immunohistochemistry (Table 3 and Figure 4) revealed that ALI patients surviving their hospitalization also displayed significantly higher levels of degranulation compared to non-surviving patients (2.62 ± 0.18/*hpf vs*. 1.58 ± 0.10/*hpf*, respectively).

**Table 3:** Eosinophil Degranulation Scores Derived from *EPX-mAb *Immunohistochemistry Algorithm

Pulmonary Patients		Eosinophil Degranulation Scores			Mean	±SEM
				
		PhD Graduate Student (LW)	Pathology Fellow (EJ)	Board-certified Pulmonary Pathologist (KL)		
Non-involved Lung Tissue from Otherwise "Healthy" Control Subjects		2.3	0	2.0	1.43	0.72
		
		2.0	0.8	1.9	1.57	0.38
		
		1.2	0	1.3	0.83	0.42
		
		1.4	0	1.3	0.90	0.45
		
		1.2	0	1.4	0.87	0.44
		
		1.2	0.2	1.1	0.83	0.32
		
		1.5	1	1.2	1.23	0.15
		
		1.2	0	1	0.73	0.37
		
		1.2	0.4	1.3	0.97	0.28
		
		1.2	0.2	1	0.80	0.31

Acute Lung Injury (ALI) Subjects	Surviving Patients	2.4	2.7	2.7	2.61	0.10
		
		2.0	2.2	2.5	2.23	0.15
		
		3.2	4.0	4.1	3.77	0.28
		
		1.8	2.8	2.5	2.37	0.30
		
		2.6	2.3	2.6	2.50	0.10
		
		2.4	3.7	2.9	3.00	0.38
		
		2.5	2.6	2.2	2.43	0.12
		
		1.1	1.1	1.4	1.20	0.10
		
		3.8	3.9	3.6	3.77	0.09
		
		3.1	3.2	3.3	3.20	0.06
		
		2.2	3.3	2.9	2.80	0.32
		
		1.8	1.2	1.7	1.57	0.19
	
	Non-surviving Patients	1.2	1.1	1.7	1.33	0.15
		
		1.8	2.0	1.9	1.90	0.05
		
		1.5	1.2	1.0	1.23	0.12
		
		1.1	1.2	1.0	1.10	0.05
		
		2.0	1.9	2.3	2.07	0.10
		
		2.7	2.4	1.7	2.27	0.24
		
		1.4	1.2	1.4	1.33	0.05
		
		1.4	1.4	1.3	1.37	0.03

## Discussion

Independent of any conclusions regarding our evaluation of ALI vs. control subjects, two technical observations regarding our assessments of the lung biopsies using ***EPX-mAb ***immunohistochemistry relative to ***H&E ***staining are noteworthy: **(i) *EPX-mAb ***immunohistochemistry is an easily performed assessment that provided a >40-fold enhancement to detect tissue infiltrating eosinophils. This increased sensitivity not only allowed for the greater detection of tissue infiltrating eosinophils but also corresponding increases in the speed, accuracy, and reproducibility of this determination. **(ii) *EPX-mAb ***immunohistochemistry provided a rapid and definitively quantitative assessment of eosinophil degranulation within the lung parenchyma, observable even in the absence of intact infiltrating eosinophils.

Unfortunately, studies of ALI patients are often incomplete and subject to ambiguities resulting from the broad and complex character of symptoms and contributing etiologies [2,37]. Compounding these issues are the limited sample materials that are available for analysis (e.g., lung tissue or BAL fluid), including the timing of when the samples were recovered during the course of a given patient's care. In this respect, the study presented here is subject to these very same limitations. That is, our study is of a small heterogeneous cohort of patients (*n = *20) whose disease origins and severity vary considerably. Moreover, we did not have control over the general demographics of these ALI patients nor could we dictate why, when, or where within the lung the biopsies for study were taken relative to the course of disease and/or patient treatment. The ALI subjects of this study were also not selected on the basis of a defined and standardized medical history or a regimented treatment plan. Finally, the control subjects available to us were limited and did not include healthy volunteer biopsies or ALI patients prior to any medical interventions.

Given the limitations of the ALI study group and our control subjects noted above, we were surprised at the ability of ***EPX-mAb ***immunohistochemistry to distinguish ALI patients from control subjects. Specifically, eosinophils are not considered a reliable histopathological marker of ALI (reviewed in [5,28]). Yet in a completely patient-blinded fashion that was reproducible among 3-4 independent evaluators who had no knowledge of one another's assessments, our numerical results were able to identify a group of ALI patients relative to control subjects on the a basis of both increased numbers of tissue infiltrating eosinophils and increased levels of eosinophil degranulation. Furthermore, these evaluations allowed us in a completely clinical outcome-blinded fashion to stratify the ALI patients into those surviving their hospitalization relative to the non-surviving patients. It is clear that the design and power of this study precludes us from overly provocative conclusions regarding the role of eosinophils, including their link with specific symptoms or their part in pathways that exacerbate or attenuate disease pathologies. Nonetheless, this study does suggest that ***EPX-mAb ***immunohistochemistry may represent a previously unrecognized diagnostic tool providing prognostic information for the management of ALI patients. In addition, given the paucity of available therapeutic options and discriminatory testing modalities, [1,6,8,10,19] assays detecting the release of eosinophil products (e.g., ELISA based assessment of degranulation from biological fluids such as breath condensate, intratracheal tube secretions, and/or BAL fluid) may also represent rapid and minimally invasive biomarkers of disease to assess this difficult patient population [38]. Indeed, this initial report provides the rationale for future studies of increased design and complexity to expand this link between ALI patients and their survival based on evidence of pulmonary eosinophils and tissue degranulation.

## Conclusions

The studies presented in this report have identified both significant technical insights and revelations regarding eosinophils in lung biopsy samples from control and ALI patients. These observations will likely have a direct impact on the assessment of eosinophils and their role(s) in lung diseases and, more important, may lead to previously overlooked or untried diagnostic and therapeutic strategies with which to treat these problematic patients.

• Immunohistochemical detection of EPX in lung tissue biopsies provides even a board certified pathologist (with a specialty in pulmonary diseases) a >40-fold increase in sensitivity to detect tissue infiltrating eosinophils in lung tissue sections

• ***EPX-mAb ***based immunohistochemistry allows for the unique assessment of eosinophil activation (i.e., degranulation events) within the lung even in the absence of a demonstrable eosinophil infiltrate

• The increased sensitivities afforded by ***EPX-mAb ***demonstrate that increases in lung infiltrating eosinophils and evidence of eosinophil degranulation are surprisingly characteristic features of biopsies from ALI patients relative to control subjects

• Assessments of lung biopsies from ALI patients following ***EPX-mAb ***immunohistochemical staining may provide a potential basis to identify a subset of the ~40% of ALI patients who will not survive their hospitalization.

## Competing interests

The authors declare that they have no competing interests.

## Authors' contributions

The corresponding author (JJL) had full access to all of the data reported in this study and had final responsibility for the decision to submit this report for publication. KP, LW, KL, JJL, designed research study; LW KP, CAP, EJ, PG, KL, and JJL performed research; CAP and NAL contributed new reagents/analytical tools; LW, CAP, RM, KL, and JJL analyzed data; and LW, KL, NAL, and JJL wrote the paper. All authors read and approved the final manuscript.
